# How plausible is it that PEP would be cost‐effective in sub‐Saharan Africa?

**DOI:** 10.1002/jia2.26455

**Published:** 2025-06-26

**Authors:** Geoffrey Peter Garnett, Peter Godfrey‐Faussett

**Affiliations:** ^1^ TB & HIV Team, Gates Foundation Seattle Washington USA; ^2^ LSHTM London UK

**Keywords:** cost‐effectiveness analysis, HIV, mathematical modelling, post‐exposure prophylaxis, sub‐Saharan Africa, transmission probabilities

## Abstract

**Introduction:**

Post‐exposure prophylaxis (PEP) is an efficacious HIV prevention tool when used soon after a potential exposure. Understanding the drivers of cost‐effectiveness of PEP in different contexts will likely play a role in determining local policies for providing PEP.

**Discussion:**

The cost‐effectiveness of PEP depends upon the likelihood of exposure to HIV, the transmission probability per sexual act and the efficacy of PEP, along with associated costs. The transmission probability per sex act will be greater in the first few acts in a partnership than on average across all acts owing to heterogeneity in the transmission probability between partnerships. In settings with high HIV prevalence and low treatment coverage, appropriately focused PEP is cost‐saving. As treatment coverage improves, PEP can remain cost‐effective with HIV prevalences above 15% with treatment coverage achieving 90:90:90 treatment targets. At 95:95:95 treatment levels, it is unlikely to be cost‐effective. PEP is only cost‐effective for the first few sex acts within a partnership. The cost‐effectiveness of PEP is sensitive to assumptions about the proportion of the population of partners with unsuppressed HIV, the pattern of mixing of those with unsuppressed virus, the transmission probability per sexual act, PEP efficacy, the costs of PEP and the value attached to preventing HIV acquisition. Where possible local parameters should be used in evaluating PEP cost‐effectiveness in our model.

**Conclusions:**

We illustrate the use of simple calculations to define the cost‐effectiveness of PEP. In populations where there is a high prevalence of unsuppressed HIV, PEP is likely to be cost‐effective but only if used for one off sexual encounters and the first few sex acts within a partnership.

## 1 INTRODUCTION

Despite the lack of randomized controlled trials, it is clear that efficacious antiretroviral treatment shortly after exposure to HIV—post‐exposure prophylaxis (PEP)—can prevent HIV acquisition [1]. Unfortunately, this approach to HIV control has been underrecognized, underused and poorly implemented. WHO guidelines from 2024 [2] that recommend community and pharmacy access to PEP aim to make PEP more widely available and more effective. In developing these guidelines, a systematic review of PEP costs and cost‐effectiveness generated little evidence [3]. Nonetheless, economic considerations will inform policy decisions about the wider use of PEP, and it is possible from empirical observation of HIV epidemiology and first principles to estimate the likely cost‐effectiveness of PEP and in what circumstances its use will be worthwhile.

Initial evidence of the protective effect of PEP was found in a retrospective case‐control study with PEP recommended and used to prevent occupational exposure to HIV [4]. This was expanded to use among those exposed to sexual violence, and as more efficacious and less toxic antiretroviral drugs have become available, use has expanded to broader types of HIV exposure. The currently preferred first‐line antiretroviral treatment tenofovir, lamivudine and dolutegravir (TLD) is recommended for PEP with a 28‐day course.

The cost‐effectiveness of PEP was explored in a 1998 study of the US context, where the cost of the regimen was $805 and a cost per Quality Adjusted Life Year threshold of $50,000 was used [5]. PEP was found to be cost‐effective for receptive anal intercourse, but not for insertive anal and vaginal intercourse. The only study of the cost‐effectiveness of PEP in the context of sub‐Saharan Africa, from 2023, explored community availability of TLD for treatment, oral pre‐exposure prophylaxis (PrEP) and PEP, and found that in most scenarios, it would be cost‐effective at the population level [6].

In this commentary, we show how the cost‐effectiveness of PEP can be approximated with simple equations, review the parameters for these equations and illustrate the circumstances in which PEP would and would not be cost‐effective.

## 2 DISCUSSION

### 2.1 Equations describing the cost‐effectiveness of PEP

The cost‐effectiveness of PEP will be a function of how likely someone using it was actually exposed to HIV, what the likelihood of acquiring HIV from an exposure is, how likely PEP is to avert that acquisition, the costs of PEP and the lifetime costs of HIV acquisition. The likelihood of exposure is a function of the distribution of viral load among contacts and the likelihood of transmission upon exposure is a function of the route of exposure and presence or absence of a range of co‐factors.

In assessing the cost‐effectiveness of PEP, a useful metric is the number needed to treat (NNT), that is the number of PEP uses to prevent one acquisition of HIV. Normally, the NNT is derived from a specific trial based on the number of incident events averted by the treatment [7]. However, from first principles, a general NNT can be derived from the efficacy of a treatment and the incidence of HIV acquisition, and is given by:

(1) $$NNT=1/\left(r.e\right),\;$$

where *e* is the efficacy of PEP and *r* is the risk of HIV acquisition for a particular unprotected exposure in the absence of PEP.

This risk *r* can be summarized by the equation:

(2) $$r=P.\left(1-s\right).m.\beta ,$$

where *P* is the prevalence of HIV in the sexual partner pool, *s* is the proportion of people living with HIV (PLHIV) who are virally suppressed, *m* a term representing an increased (or decreased) chance that a partner is living with HIV and unsuppressed based on patterns of risk behaviour and *β* represents the transmission probability per act for HIV. We assume there is no transmission from those who are virally suppressed. More details of the complexity summarized by these parameters are presented below.

The cost per HIV acquisition averted C is given by: C = NTT.k, where k is the cost per PEP episode.

The cost per acquisition averted can be compared with the costs of treating an HIV case and the disability‐adjusted life‐years (DALY) associated with a case to determine whether PEP is cost‐saving or cost‐effective at a given threshold. The cost per DALY averted *A* is given by:

(3) $$A=\left(C-T\right)/D$$

where *T* is the lifetime cost of treating someone for HIV and *D* is the DALYs associated with an HIV acquisition.

The impact in terms of number of HIV acquisitions averted *H* is given by:

(4) H=N.r.e,

where *N* is the number of PEP users, which is determined by the product of the population at risk, the proportion using PEP and the frequency of use per year. Note that impact is not a function of cost or cost‐effectiveness, but of the budget impact, which is the product of number of people using PEP and the cost per episode of PEP.

The cost‐effectiveness of PEP calculated above is only an approximation, as it does not account for changes in HIV prevalence associated with a PEP programme, or the knock‐on benefits from each new person not acquiring HIV averting further acquisitions among their contacts.

### 2.2 The prevalence of unsuppressed PLHIV in the partner pool

HIV prevalence by age and sex is estimated by UNAIDS [8]. These prevalence estimates can be combined with estimates for viral suppression among PLHIV from routine clinical data or population‐based surveys [9]. Even when treatment coverage is high, there can be moderately high prevalences of unsuppressed PLHIV [10].

### 2.3 HIV viral loads and the HIV transmission probability

Empirical data suggest that HIV is not transmitted when the HIV viral load is below 1000 copies per ml [11]. In a review of surveys [8], the average viral load of those unsuppressed was around 10,000 copies per ml. If viral load testing uses a threshold of 1000 copies per ml, most of those with viraemia will be able to transmit, whereas if the more precise thresholds of 50 and 400 which are now sometimes used in clinical management are used, then not all those classified as unsuppressed would be able to transmit the virus. If we define the community viral load as the proportion of the population with an unsuppressed virus (rather than the mean level of viraemia), then the community viral load is the product of the prevalence of HIV and the proportion of unsuppressed PLHIV (*P*.(1‐*s*) in Equation 2). The average transmission probability per act of HIV‐1 measured in retrospective and prospective studies of heterosexual couples is around 1 in a thousand [12, 13, 14] with an order of magnitude higher probability for anal intercourse, including among men who have sex with men (MSM) [15]. However, when considering one‐time sex acts, particularly with new sexual partners, this average value is misleading [12, 16, 17].

There is heterogeneity in the risk of transmission driven by many variables, including type of sex (receptive and insertive, vaginal and anal), viral load of the partner, the presence of genital ulcer disease, male circumcision status, age and sexual maturity [12, 13, 14]. This heterogeneity between partnerships meant that initial studies of HIV transmission found no correlation between the number of sexual exposures within a partnership and HIV transmission [18]. This can be explained by a majority of HIV serodifferent sexual partnerships involving a low risk of transmission per act with only a minority of such partnerships involving a high risk of transmission [16, 17]. The average transmission probability measured from stable partnerships records many acts with no transmission in low‐risk partnerships and few acts before transmission in high‐risk partnerships, biasing estimates to a lower transmission probability.

Boily and colleagues [13] found transmission probabilities to be greater 0.0087 for females to males and 0.0019 for males to females in low‐ and‐middle‐income countries (LMICs). There was much heterogeneity in estimates, with being uncircumcised increasing the probability three‐ to eight‐fold and an early‐stage infection increasing risk by nine‐fold compared to the asymptomatic stage.

**Figure 1:**
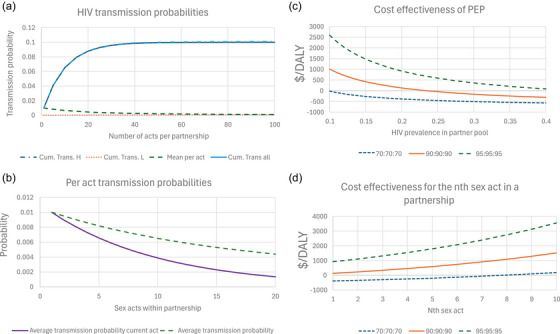
**The relationship between unsuppressed HIV virus, the per act HIV transmission probability and the cost‐effectiveness of post‐exposure prophylaxis PEP**. (a) The HIV transmission probability as a function of the number of condomless sexual acts in a partnership when 10% of partnerships have a transmission probability per act of 0.1 and 90% of 0.0001. The transmission probability among the 10% of high‐risk partnerships, 90% of low‐risk partnerships and all partnerships is shown along with the average transmission probability per act. (b) The average transmission probability per act and the transmission probability in the current act for the *n*th condomless sex act in a partnership is shown when 10% of partnerships have a transmission probability of 0.1 and 90% of 0.0001. (c) The cost‐effectiveness of an episode of PEP used after the first condomless sex act in a partnership as a function of HIV prevalence in the partner pool. The cost in dollars per DALY averted is shown when treatment coverage has suppressed virus in 34.3%, 72.9% and 85.7% (in the language of UNAIDS targets 70:70:70, 90:90:90 and 95:95:95). The transmission probability per sex act is 0.010009, the mixing parameter *m* = 1.5, efficacy is 70%, the cost per episode of PEP is $10, the lifetime cost of HIV treatment is $15,0000 and each new HIV acquisition incurs 20 DALYs. (d) The cost‐effectiveness of an episode of PEP used in the *n*th condomless sex act within a partnership for three levels of viral suppression.

A model of transmission is illustrated in Figure 1a where in 10% of partnerships the transmission probability is 0.1 per act and in 90% of partnerships it is 0.00001 per act. The first act has an average transmission probability 0.01009. The average transmission probability per act falls as acts accumulate, after four acts, the average per act transmission probability is 0.0087, as observed for female‐to‐male transmission in LMICs, and is 0.0019 after 53 acts as observed for male‐to‐female transmission in LMICs. Because transmission is occurring early in risky partnerships and not in other partnerships, the average transmission probability falls as acts increase. The average transmission probability per current act across partnerships falls faster than the average across partnerships for cumulative acts (Figure 1b); starting at over 0.01, it is around 0.004 after 10 acts and 0.0014 after 20 acts in the partnership.

### 2.4 Patterns of mixing

The likelihood that a one‐time sexual contact is with someone living with HIV who has an unsuppressed virus is a function of the proportion of short‐lived sexual partnerships among those virally unsuppressed. This depends on not only the fraction of the population that is viraemic but also whether they are more or less likely to be engaged in such partnerships, which depends on the number of such partnerships they form and the pattern of sexual mixing they have with groups seeking PEP. The value *m* in Equation (2) weights the likelihood that a contact is viraemic. If mixing is random, then the probability of a new partnership being with someone who is virally unsuppressed is given by $P\left(1-s\right).f_{v}/F$ where *P(1‐s)* is the proportion of the population virally suppressed, *f_v_* is the rate of new partnership formation by those virally suppressed and F is the mean rate at which all people in the partner pool form partnerships.

In addition, there is an influence of how people mix sexually according to social, cultural and demographic variables. It is likely that those virally unsuppressed have characteristics correlated with some of these variables. Patterns of sexual mixing can vary on a scale from assortative (like with like) through random, matching the proportion of partnerships created by a group, to disassortative (like with unlike). Using a parameter, *ε*, to define the assortativeness of mixing where *ε* = 0 is assortative mixing and *ε* = 1 is random mixing, the likelihood a partner being viraemic is $\varepsilon P\left(1-s\right)f_{v}/F$ [17]. This allows us to define *m* as $m=\varepsilon f_{v}/F$ since the fraction of the population virally unsuppressed is already included in the numerator of Equation (2).

### 2.5 Efficacy of PEP

In the original case‐control study where zidovudine was used for PEP, its efficacy was estimated to be 79% [4], in a subsequent study of non‐occupational exposure to HIV among MSM in Brazil, efficacy seems to have been 88% [19]. In a review of other clinical studies [1] of 2692 PEP courses, only one seroconversion is recorded in someone with multiple high‐risk exposures before and after starting PEP. Efficacy will depend on how soon after exposure PEP is used and whether the course is completed. In our analyses, we assume a PEP efficacy of 70% but also look at the influence of greater or lesser efficacy.

### 2.6 Costs

In low‐income countries, there have been few studies of the cost of PEP. In a paper modelling, the community availability of TLD for PEP, PrEP and treatment, Phillips and colleagues [6] assumed a cost of PEP of $19 based on 3 months of TLD. This assumed more than 28 days of the drug and did not measure the costs of delivery. In a pilot study of PEP distribution by pharmacists in Kenya, the financial cost of PEP to the provider was $9.34 per client among 162 clients [20].

The costs of treatment vary greatly across countries and depend on the costs that are included, and for a lifetime of treatment, the discount rate is assumed. For ease of illustration, we assume that treatment costs $500 per person per year and is needed for 30 years, adding to $15,000 for a lifetime. The DALYs associated with an HIV acquisition depend upon the age at which the infection is acquired, the years of life lost by those acquiring infection and the disability weight attached to living with HIV.

## 3 RESULTS

The cost‐effectiveness of PEP when used in the first condomless sex of a partnership is illustrated (Figure 1c) as a function of HIV prevalence for three different treatment levels: 70:70:70, 90:90:90 and 95:95:95. The numbers here represent the percentage of PLHIV diagnosed, the percentage of those diagnosed in a treatment programme and the percentage of those “on treatment” who are virally suppressed. These levels equate, respectively, to 34.3%, 72.9% and 85.7% virally suppressed. PEP is more cost‐effective when treatment coverage is lower, being cost‐saving when HIV prevalence is 1% and viral suppression is only 34.3%. At 95:95:95, it only becomes cost‐effective at the $500 per DALY threshold at 27% prevalence. It should be remembered that the relevant prevalences determining whether PEP is warranted are for the local partner pool, so even if average treatment coverage is high, it might still be low in groups from which partners are selected, such as MSM, sex workers or particular demographic groups.

The cost‐effectiveness of PEP declines as it is used over more condomless sex acts in a partnership or in later condomless sex acts in a partnership. Figure 1d illustrates the cost‐effectiveness of PEP in the *n*th sex act in a partnership when HIV prevalence in the partner pool is 20% for the three treatment coverage levels. When 72.9% of PLHIV are suppressed, the cost‐effectiveness for the first sex act is $128/DALY but it has risen over $500/DALY by the fifth sex act.

This has implications for the design and messaging around a PEP programme. It is most likely to be cost‐effective if PEP is used by those who have had a one off condomless sexual encounter, or very few condomless sex acts with a new partner, and not cost‐effective if used occasionally by those in longer‐term partnerships. In such partnerships, the use of PEP in the first few unprotected acts could be cost‐effective.

Using the equations, one can explore the sensitivity of results to parameter values. For example, anchoring on 20% prevalence, 72.9% viral suppression, *m* = 1.5, transmission per act = 0.010009, efficacy of 70%, costs of PEP of $10 per episode, lifetime treatment costs of HIV of $15,000 and 20 DALYs incurred for each HIV acquisition, the cost‐effectiveness of PEP is $128 per DALY. A decrease in prevalence to 10% changes this to $1006 per DALY, and an increase in prevalence to 30% changes it to −$165/DALY.

Impact is a linear function of the number of people using PEP in the right context. Using the anchor assumptions above, the number of PEP episodes for the first unprotected acts to prevent one HIV acquisition is 1756 (so the cost per acquisition averted is $17,566). PEP use 10,000 times would prevent 5.7 HIV acquisitions.

## 4 CONCLUSIONS

It can be seen how the local context of HIV prevalence, sexual mixing, the rapidity with which PEP is accessed, and the costs of PEP, HIV treatment and the burden of an HIV acquisition greatly influence whether PEP is cost‐effective. Estimates of unsuppressed virus in potential partners, as illustrated by Joseph et al. [10], could be used by decision‐makers to assess the value of a PEP programme.

Our findings diverge from Phillips and colleagues [6], who fitting a complex model to epidemic trends found PEP cost‐effective in most scenarios. Our results allow for an explicit exploration of the role of parameters, whereas Phillips and colleagues derive scenarios fitting to quite high levels of HIV incidence, largely because the data include studies dating back to 2017, while incidence has recently fallen sharply in the region. Their median model estimate for incidence of 0.5% across both sexes is higher than the current UNAIDS estimates for all countries in the region.

In general, high HIV prevalence or low levels of viral suppression in particular partner groups make a PEP programme worthwhile. However, to be cost‐effective, such a programme should focus on the first few unprotected sex acts within partnerships. For efficacy and impact, making PEP easily accessible is warranted, but messaging about who will benefit from PEP should focus on one‐time sexual encounters and the first few condomless sex acts with new partners.

## COMPETING INTERESTS

GPG is an employee of the Gates Foundation and PG‐F has received Grants from the Gates Foundations.

## AUTHORS’ CONTRIBUTIONS

The commentary was co‐conceived by PG‐F and GPG. GPG defined and explored the calculations. GPG and PG‐F both drafted the commentary.

## FUNDING

The was no specific funding for this commentary.

## DISCLAIMER

The views in this commentary are those of the authors and do not necessarily represent the position of the Gates Foundation.

## Data Availability

Data sharing is not applicable to this article as no datasets were generated or analysed during the current study.
